# Venom immunotherapy protocols in the pediatric population: how to choose?

**DOI:** 10.3389/fped.2023.1192081

**Published:** 2023-09-07

**Authors:** Francesca Saretta, Mattia Giovannini, Benedetta Pessina, Simona Barni, Giulia Liccioli, Lucrezia Sarti, Leonardo Tomei, Camilla Fazi, Francesco Pegoraro, Claudia Valleriani, Silvia Ricci, Chiara Azzari, Elio Novembre, Francesca Mori

**Affiliations:** ^1^ Pediatric Department, Latisana-Palmanova Hospital, Azienda Sanitaria Universitaria Friuli Centrale, Udine, Italy; ^2^ Department of Health Sciences, University of Florence, Florence, Italy; ^3^ Allergy Unit, Meyer Children's Hospital IRCCS, Florence, Italy; ^4^ Immunology Laboratory, Meyer Children's Hospital IRCCS, Florence, Italy; ^5^ Immunology Unit, Meyer Children's Hospital IRCCS, Florence, Italy

**Keywords:** precision medicine, hymenoptera venom allergy, protocols, pediatrics, venom immunotherapy

## Introduction

Anaphylaxis from insect stings is one of the main causes of anaphylaxis in adults (1) and in children (2). Venom immunotherapy (VIT) is, to date, the only available long-term, natural history-modifying treatment for Hymenoptera venom allergy (HVA), and it is currently recommended mostly for sensitized patients with a history of systemic reactions (SR) after insect stings (1–3).

This recommendation is also indicated in the pediatric age (4), in which the risk of future reactions following a previous moderate-to-severe field sting is approximately 32% in untreated children (*p* = 0.007) (5). As for adults, in some specific pediatric cases, VIT can be suggested even more in the presence of risk factors (e.g., increased risk of a sting as in beekeepers' children, impaired quality of life, such as in the case of anxiety, remoteness from the emergency department) (4). In children with reported large local reactions (LLR, a swelling >10 cm and lasting >24 h), VIT is generally not recommended (5, 6).

Over the decades, VIT has proved to be safe and effective for all ages and for different HVA, providing 77%–84% effectiveness in the case of honeybee allergy and up to 91%–96% in vespid allergy (3).

In 1925, Braun et al. (7) described the first attempt of VIT with honeybee venom obtained from the insect's whole body. This type of therapy was used until the 1970s, when the first report with whole venom (8) and the first randomized controlled study (9) were published by Busse et al. and Hunt and al., respectively. In 1974, Lichtenstein et al. (10) performed the first desensitization with honeybee venom in pediatrics, in a four-year-old boy who was previously treated with whole-body extract without efficacy. In a period of two months, after showing repeated SR, the child reached the maintenance dose of 100 mcg, and he showed no SR at an in-hospital sting challenge.

One of the first studies on pediatric ages was conducted by Chipps et al. (11) on a group of 44 children (4–14 years) with a conventional protocol. The maintenance dose of 100 mcg was reached in six weeks, with a “modified-rush” build-up on day one (from 0.01 to 1 mcg). In this cohort, three (6.8%) children presented a SR, and only one was severe enough to require adrenaline treatment. An in-hospital sting challenge was conducted 15 weeks after starting VIT, showing only one reaction in a child with low IgG titers, demonstrating the efficacy and safety of this procedure in children.

In 2017, an extensive systematic review and meta-analysis was published on VIT by Dhami et al. (12). The authors concluded that VIT reduced the risk of SR (odds ratio, OR = 0.08, 95% confidence interval, CI 0.03–0.26) and improved quality of life (risk difference = 1.41, 95% CI 1.04–1.79). Adverse effects were reported in both the build-up and maintenance phases, but most of them were mild, and no fatalities were recorded. Among these studies, there were only three specifically evaluating VIT in childhood (5, 13, 14) and they were all considered with a low-moderate quality assessment.

Similar results were observed in a Cochrane review published by Boyle et al. in 2012 (15). VIT improved quality of life (mean difference in favor of VIT 1.21 points on a 7-point scale, 95% CI 0.75–1.67) and prevented both LLR (relative risk, RR = 0.41, 95% CI 0.24–0.69) and SR (RR = 0.10, 95% CI 0.03–0.28). VIT was generally well tolerated, with a risk of SR of 9.3% in treated patients vs. 0.7% in the placebo group, higher for honeybee venom (14.2%) than for wasp venom (2.8%).

In clinical practice, tests becoming negative happens only for a small percentage of patients (16). Studies recommend performing VIT for at least 3–5 years and maintaining a follow-up after suspending it (17). In specific cases, it may be suggested to continue VIT for more than 5 years, such as in the presence of specific risk factors like SR from field sting during VIT (4).

Venom immunotherapy (VIT) is, to date, the only available long-term treatment for HVA. Recommendations provided by scientific societies may also be applied to childhood, though no international pediatric-specific documents have been published for the management of HVA. In the last years, despite the huge amount of literature on VIT being produced, most of the studies evaluate only adult populations. Some of these also include children, but often, pediatric data are not separately analyzed. In the most recent years, some scientific groups have specifically studied the indications, safety, and efficacy of VIT in childhood. Nonetheless, it is still difficult to compare those studies since they analyze different VIT regimens in heterogeneous populations. In this review, we aim to dissert and evaluate relevant articles with specific data on VIT in pediatric ages.

## Materials and methods

We performed a literature search in Medline through PubMed using default keywords related to pediatric Hymenoptera Venom Allergy and Allergen Immunotherapy. Original studies and review articles, with a focus on meta-analyses and randomized controlled trials in English, were identified up to February 1st, 2023.

## Results

VIT can be carried out with different protocols, all of which have already been proven safe and effective in pediatric ages. VIT involves a build-up phase with progressively increasing doses (starting from 0.001 mcg to 0.1 mcg) of the chosen venom until the maintenance dose is reached, which is usually 100 mcg (18, 19). A starting dose of 1 mcg may be considered safe as well (20).

As underlined by Golden (21), there is no consensus on the actual duration of the build-up phase, according to existing literature, but the main build-up phase protocols are usually defined (1) conventional: months, (2) clustered or semi-rush: weeks, (3) rush or ultra-rush: days.

During maintenance, the achieved dose should be injected every four weeks in the first year and then every six to eight weeks in the following years, although some authors considered 12-week or even longer intervals between doses safe (22–24). A higher maintenance dose at 150–200 mcg may be prescribed in high-risk patients, e.g., if a patient had a SR at re-sting while on VIT (3, 25), whereas a lower dose (50 mcg) has been suggested sufficient for children (26–28).

Especially in some countries, as highlighted in an editorial by Golden (29), the protocol choice may rely more on healthcare system organization than on scientific or clinical reasons. Rush/ultra-rush protocols are usually preferred to concentrate evaluations and therapies in a few days, in countries where healthcare is provided in specialized centers, and upon the patient's request or need (e.g., rapid achievement of maintenance dose, low compliance with conventional protocol). Even though literature has already proven the safety and efficacy of rush and ultra-rush protocols (30), conventional and clustered protocols seem to be generally preferred in clinical practice. In a survey conducted in the United States (29, 31), most allergists preferred conventional (64%) compared to eight-week (31%) and rush protocols (5%). In the United Kingdom, allergists prefer conventional protocols (92%) while only 25% of respondents have ever used clustered or rush/ultra-rush protocols (32). In another survey, Martínez-Cañavate et al. (33) analyzed one of the largest groups of children treated with VIT in Spain. In most cases, a conventional protocol was chosen (68.2%), followed by rush (18.5%), clustered (11.8%), and ultra-rush (1.5%).

A summary of pediatric studies reporting VIT protocols is shown in Table 1, and some examples are discussed below.

**Table 1:** Summary of pediatric studies reporting VIT protocols.

Study	Population	Protocol	Reaction as per		Notes
			patient	injection	
Conventional protocols					
Albuhairi et al. (34) Ann Allergy Asthma Immunol 2018 ONLY CHILDREN	78 children (5–18 y/o) M: 60—F: 18 8 y/o at sting 9 y/o at VIT 10.4% mild cutaneous 5.3% LLR (≥5 cm) 72.7% moderate SR 11.6% severe SR 75.3% treated with mixed venom extracts	Build-up: weekly increases starting from 0.1 mcg (8–10 weeks) Maintenance: 100 mcg (every 4–6 weeks)	9% SR (7/78) - 5/7 grade 1 - 2/7 grade 2 6/7 immediate (≤30 min) 1/7 delayed 4/7 during build-up 3/7 during maintenance	0.2% SR/injection (3564 doses) 5/7 required therapy No adrenaline None had history of severe SR at sting	Risk factor: male sex (for moderate/severe field SR) (*p* = 0.008) No risk factors for VIT-SR (atopy, age, sex, asthma, venom) 21 (27%) re-stung during VIT (one in build-up): - 1 (5%) moderate SR (hives and cough) - 12 (57%) local reactions - 8 (38%) no reactions
Gür Çetinkaya et al. (35) Ann Allergy Asthma Immunol 2018 ONLY CHILDREN	107 children M: 77—F: 30 median age 10 y/o At least 1 systemic reaction after sting 75.7% wasp 23.3% honeybee 0.9% both venoms 17 did not complete VIT	Build-up: weekly increases starting from 3 to 8 mcg (6 months) Maintenance: 100 mcg (every 4–6 weeks for 5 years)	52/107 had AR 37.4% local reactions 4.7% LLR (2 during build-up, 2 at maintenance, 1 not specified) 6.5% SR (7/107) - 4/7 grade 1–2 - 2/7 grade 3 - 1/7 grade 4 3/7 during build-up 4/7 during maintenance	0.17% SR/injection (5671 doses) Local 1.6% LLR 0.14%	Risk factor: asthma in multivariate analysis (*p* = 0.016) Wasp = more local reactions; honeybee = more severe systemic reactions. SR during honeybee VIT 16%; SR during wasp VIT 3.7% (*p* = 0.031). 33/68 contacted (48.5%) were re-stung averagely in a 34-month interval from VIT starting date: - 4 (12%) SR (mild, grade 1–2) - 1 (3%) LLR - 19 (58%) local - 9 (27%) no reactions
Chipps et al. (11) J Pediatr 1980 ONLY CHILDREN	44 children (4–14 y/o) mean age 9.6 y/o 98% at least cutaneous 48% at least respiratory 9% hypotension 27% life threatening SR 50% vespid venom 11.3% honeybee venom 38.6% > 1 (vespid +/− honeybee	Build-up: weekly increases starting from 0.1 to 1 mcg on day 1 (6 weeks) Maintenance: 100 mcg (every 1–4 weeks)	6.8% SR (3/44) 27% LLR (12/44)	0.3% SR/injection LLR 2.5% 1 required adrenaline	Only 1 reaction at hospital sting challenge (20 children), in a child with low venom-specific IgG titers
Clustered and semi-rush protocols					
Konstantinou et al. (28) Pediatr Allergy Immunol 2011 ONLY CHILDREN	54 children M: 29—F: 25 mean age 9.5 y/o 77.8% grade 3 13% grade 3/4 9.2% grade 4 1 lost to follow-up	Modified-cluster build-up: cumulative dose on day 1 = 0.6111 mcg to 50 mcg in 31 days Maintenance: 50 mcg every 4 weeks for 1° year every 5 weeks for 2°–3° every 6 weeks for 4°–5°	3.8% SR (2/53): maintenance dose was increased to 100 mcg 51/53 who completed VIT showed no side effects apart from mild reactions at injection site 75.5% needed antihistamine therapy, more often during build-up In the 2 with SR: both allergic to honeybee; -1 boy with generalized urticaria during build-up -1 girl with rhinitis, wheezing, dyspnea, hypotension and asthenia		53 completed 5 years (1 lost to follow-up without reported reactions) At re-sting (21/51 with 50 mcg maintenance): no SR - 10/21 during VIT - 11/21 after VIT was completed
Carballada Gonzales et al. (36) Allergol Immunopathol 2009 ONLY CHILDREN	21 children (4–16 y/o) M: 13—F: 8 mean age 11.8 y/o 9.5% grade 1 28.6% grade 2 57.1% grade 3 4.8% grade 4 80.9% honeybee 19.1% hornet 85.7% completed VIT	Semi-rush build-up: 1–2 weekly injection of 9 increasing doses Maintenance: 100 mcg VIT for 5 years or until negative test	5 adverse reactions all for honeybee venom 3 local 2 SR (9.5%) no LLR 1 adrenaline 1 antihistamines and BD VIT resumed with previous tolerated dose		7 (33.3%) re-stung (5 during maintenance): - 4/7 no reactions - 3/7 mild local reactions
Rush and ultra-rush protocols					
Glaeser et al. (37) Clin Mol Allergy 2022 ONLY CHILDREN	19 children median age 9 y/o All with history of honeybee anaphylaxis 16% grade 1 42% grade 2 42% grade 3 0% grade 4	Rush build-up: 1 mcg to 100 mcg in 3 days	SR 15.8% (*n* = 3) -1 grade 1 -2 grade 2 Local reactions 58.8% All reactions on day 2 (1 at 40 mcg and 2 at 80 mcg)		All reached maintenance dose (100 mcg) and entered maintenance phase, no further reactions in 2 years
Stoevesandt et al. (38) Allergy Asthma Clin Immunol 2017 CHILDREN vs. ADULTS	71 children (7–17 y/o) M: 45—F: 26 median 14 y/o 981 adult controls SR 9.9% children vs. SR 26.5% adults (*p* = 0.001) Honeybee allergy: 32.4% children vs. 14.7% adults (*p* < 0.001) Lower baseline tryptase levels in children (*p* = 0.014)	Rush build-up: 0.1 mcg to 100 mcg in 3 or 5 days Maintenance: 100 mcg	SR 6.9% children vs. 2.5% adults (*p* = 0.046) Children anaphylaxis (*n* = 5): - 2 honeybee: grade 1 and grade 2 - 3 wasp: all grade 1 No adrenaline needed, all reached 100 mcg dose LRR children 9.7% vs. 6.8% adults (*p* = ns)	72 cycles in children (1 child only received double VIT for both honeybee and wasp) 1029 cycles in adults 728 injections among children 10217 injections among adults	Regression model VIT honeybee (OR 2.25, *p* = 0.039) and build-up in 5 days vs. 3 days build-up (OR 2.64, *p* = 0.011) associated with increased risk for VIT-SR Higher rate of VIT anaphylaxis in children due to higher prevalence of honeybee venom allergy in children
Nittner-Marszalska et al. (39) J Investig Allergol Clin Immunol 2016 CHILDREN vs. ADULTS	134 children 4-17 y/o M: 94—F: 40 mean age 12.6 y/o 207 adult controls 9.0% grade 1 17.9% grade 2 47.8% grade 3 25.3% grade 4 48.5% wasp (M: 66.2%) 51.5% honeybee (M: 73.9%)	Ultra-rush build-up: 0.1 mcg to 101.1 mcg in 3.5 h Maintenance: not described	SR 3.7% children (*n* = 5) vs. 7.7% adults (*p* = ns) SR to honeybee venom: 7.2% children vs. 21.4% adults (*p* = 0.034) All 5 children were allergic to honeybee: - 4/5 had grade 4 first SR - 4/5 had grade 3 VIT-SR All during build-up		Children: - total and specific IgE higher than adults (*p* < 0.001) - lower median tryptase than adults (*p* = 0.009) Increased VIT-SR risk if first reaction was grade 4 compared to grade 3 (*p* = 0.016); grade 1–2 field reactions did not show any VIT-SR Increased VIT-SR risk in children allergic to honeybee compared to wasp allergy (*p* = 0.058). No difference adults/children who experienced VIT-SR with respect to asthma, atopy, severity previous reaction, skin tests or sIgE
Steiss et al. (40) J Aller Ther 2013 ONLY CHILDREN	90 children (4–17 y/o) M: 56—F: 34 mean age 9.3 y/o 7.8% grade 1 18.9% grade 2 64.4% grade 3 8.9% grade 4 57.8% wasp 42.2% honeybee	Modified ultra-rush build-up: day 1: 7 doses up to 80 mcg day 2: 100 mcg Maintenance: 100 mcg booster at week 1, week 3, then every 4–6 weeks	2.2% SR (*n* = 2), both honeybee 1 mild dyspnea 1 urticaria 16.7% local maximum 15 cm 22.2% LLR maximum 20 cm Median: 40–80 mcg no difference honeybee/wasp	0.002% SR (720 injections) No adrenaline IV antihistamines or corticosteroid +/− inhaled BD	
Kohli-Wiesner et al. (41) J Allergy 2012 ONLY CHILDREN	94 children (4–15 y/o) M: 70—F: 24 mean age 10.4 y/o All grade 2–4 65% honeybee 35% wasp 8.5% both venoms 102 ultra-rush (double allergy counted twice) 15 group A 4–8 y/o 60 group B 8–12 y/o 27 group C 12–15 y/o	Ultra-rush build-up: 0.1 mcg to 110 mcg cumulative dose in 210 min in at least 6 injections Booster on day 7: two 50 mcg doses with a 30-min interval Week 3 and 7: 100 mcg	16% SR (*n* = 16) - 6/16 (37.5%) grade 1 - 2/16 (12.5%) grade 2 - 5/16 (31%) grade 3 - 3/16 (19%) unclassifiable No SR in group A 18% SR in group B 19% SR in group C SR F 29% vs. M 12% (*p* = 0.034) More SR reaction at 50 mcg No adrenaline No antihistamine premedication		SR honeybee 20% vs. 8% wasp (*p* = ns)
Steiss et al. (42) Allergy Asthma Proc 2006 ONLY CHILDREN	43 children - 16 honeybee 7 M—9 F - 27 wasp 19 M—8 F mean age 9.5 y/o grade 1: 1 honeybee, 0 wasp grade 2: 12 honeybee, 15 wasp grade 3: 3 honeybee, 12 wasp grade 4: 0 honeybee, 0 wasp	11.6% (*n* = 5) ultra-rush build-up: day 1: 9 doses up to 100 mcg in 24 h day 2: 100 mcg vs. 88.4% (*n* = 38) modified ultra-rush build-up: day 1: 7 doses up to 80 mcg in 3 h day 2: 100 mcg Maintenance: 100 mcg booster at week 1, week 3, then every 4–6 weeks	No SR 58.1% no reaction 16.2% local maximum 15 cm 25.6% LLR maximum 20 cm median 40–80 mcg no difference honeybee/wasp	38 children with 304 injections (8 each) No therapy needed Reduction dose needed	
Birnbaum et al. (43) Clin Exp Allergy 2003 CHILDREN vs. ADULTS	51 children mean age 9.2 y/o - 33.3% honeybee - 74.5% yellow jacket - 19.6% wasp 207 adults age 40.62 y/o 195 single VIT 59 double VIT 4 triple VIT	Ultra-rush build-up: in 3.5 h day 1: 6 doses, 0.1–40 mcg day 15: 2 doses, 50 mcg day 45: 1 dose, 100 mcg Cumulative dose 101.1 mcg Maintenance: 100 mcg monthly	13.9% SR (*n* = 36, 33 on day 1, 2 on day 15, 1 on day 45) SR: 10.8% children vs. 11.2% adults (*p* = ns) Association between field severity and VIT severity children (*p* = 0.025) adult (*p* = 0.016) Most reactions at 10–40 mcg 2 required adrenaline		No late reactions No severe cardiovascular reactions in children Honeybee venom and prior sting grade 3–4 increased risk of VIT-SR No relation between IgE and risk of VIT-SR Age and sex not a risk factor for VIT-SR More positive skin test risk factor for VIT-SR
Comparison between different protocols					
Johnston et al. (44) J Paediatr Child Health 2022 ONLY CHILDREN	14 children 6 URVIT (median age 7.3 y/o, M: 5) 8 CVIT (median age 8 y/o, M: 6) All with history of honeybee anaphylaxis	URVIT build-up: in 3.5 h day 1: 6 doses, 0.1–40 mcg day 15: 2 doses, 50 mcg day 45: 1 dose, 100 mcg Cumulative dose 101.1 mcg CVIT build-up: from 0.1 mcg to 100 mcg, weekly increases for 14 weeks	SR: URVIT 16.6% vs. CVIT 25% 11/14 none or local reaction only	149 injections 1 required adrenaline (CVIT group)	URVIT completed updosing protocol in shorter time (6 vs. 14 weeks), had fewer hospital visits (3 vs. 12) and fewer injections (9 vs. 12). Cumulative distance savings for the URVIT group
Confino-Cohen et al. (45) J Allergy Clin Immunol Pract 2017 ONLY CHILDREN	127 children (2–18 y/o) M: 97—F: 30 mean age 10.56 y/o 66% rush 34% conventional 3.1% grade 1 89.8% grade 2 7.1% grade 3 RVIT honeybee 83.3% CVIT honeybee 69.7% (*p* = 0.015)	Rush build-up: 0.05 mcg to 100 mcg in 3 days Conventional build-up: 0.05 mcg to 100 mcg in 17 weeks Maintenance: 100 mcg	SR during build-up: RVIT 19.0% vs. CVIT 23.2% (*p* = ns) - grade 1: RVIT 81.3% vs. CVIT 85% - grade 2: RVIT 18.7% vs. CVIT 20% Adrenaline use: RVIT 4.7% vs. CVIT 9.3% (*p* = ns) SR at maintenance: RVIT 9.3% vs. CVIT 14.8% (*p* = ns) -grade 1: RVIT 100% vs. CVIT 50% -grade 2: RVIT 0 vs. CVIT 50% No grade 3 reactions		FU available for 102/127 children (some did only build-up phase then FU by family physician) 75 RVIT and 27 CVIT Maintenance dose reached in 90.7% CVIT vs. 98.8% RVIT (*p* = 0.04) Less SR for honeybee-rush vs. honeybee-conventional (RVIT 18% vs. 30% CVIT, *p* = ns) Age group 2–6 years: 13 RVIT (all honeybee) 8 CVIT (6 honeybee, 2 also wasp/yellow jacket) Mostly grade 2 reactions at field sting and mostly grade 1 during VIT (reduction of severity) No adrenaline needed Maintenance dose reached in 92.3% RVIT vs. 100% CVIT
Martínez-Cañavate et al. (33) Allergol Immunopathol 2010 ONLY CHILDREN	175 children (2–17 y/o) M: 135—F: 40 mean age 9.9 y/o 17% beekeeper son 68.9% previous sting 83.9% local reaction 8% anaphylaxis Compared VIT vs. prevention vs. clinical manifestations	135/175 VIT for average duration of 3.5 years CVIT 92/135 RVIT 25/135 cluster 16/135 URVIT 2/135 45 honeybee 45 Polistes 39 wasp 2 wasp + Polistes	0.4% SR (*n* = 6) - wasp 2/6 grade 3 - Polistes 1/6 grade 3 - honeybee 3/6 grade 1–3 2/92 conventional 1/25 rush 1/16 cluster 2 not specified 25% local reactions (*n* = 35) - wasp 7/35 - Polistes 14/35 - honeybee 14/35		Most of VIT are prescribed for local reactions
Treated vs. untreated					
Golden et al. (5) N Engl J Med 2004 ONLY CHILDREN	1033 children (356 VIT, mean duration 3.5 years) 226 LLR (no VIT) 462 mild skin (110 VIT) 345 moderate-severe (246 VIT) 512 (50%) children (mean age 8 y/o) answered survey: VIT: 46% no VIT: 53%		SR: VIT: 3% (2/64) no VIT: 17% (19/111) (*p* = 0.007) Untreated at re-sting: LLR: 7% SR Mild: 13% SR Moderate-severe: 32% SR		Similar rate of sting between VIT and no VIT (*p* = 0.22) History of moderate-severe reactions has a higher rate of reaction if not treated (32% vs. 5%, *p* = 0.007) Frequency of SR decreases over the 20-year observation period (*p* = ns) SR rate in untreated with mild skin reactions is lower than the rate in untreated with moderate-severe reactions (*p* = 0.05) Long protection of VIT even 10 to 20 years after therapy is suspended
Omalizumab pretreatment					
Droitcourt et al. (46) Allergol Int 2019 ONLY CHILDREN	3 teenagers with severe anaphylaxis 15 y/o M honeybee 12 y/o M wasp 14 y/o M honeybee	rush VIT with omalizumab pretreatment	Dyspnea and generalized urticaria Anaphylaxis Dyspnea, stridor and generalized urticaria		All tolerated The same department had treated 90 children with 5.6% of SR during rush build-up phase

AR, allergic reactions; BD, bronchodilators; CVIT, conventional venom immunotherapy; F, female; FU, follow-up; IM, intramuscular; LLR, large local reactions; M, male; ns, not significant; RVIT, rush venom immunotherapy; SR, systemic reactions; URVIT, ultra-rush venom immunotherapy; VIT, venom immunotherapy; vs.: versus; y/o, years old.

### Conventional protocols

In a group of 78 children with a history of SR (mild 10.4%, moderate 72.7%, severe 11.6%) studied by Albuhairi et al., treatment with a conventional protocol resulted in 9% of SR during VIT (34). None of those were severe, with a 0.2% SR rate per injection. Twenty-one children were re-stung during VIT, and 12 (57%) showed a local reaction, one (5%) showed a SR, and eight (38%) showed no reactions at all. No statistically significant risk factor for SR prediction has been identified (e.g., age, atopy, gender, asthma, or type of venom).

A similar result was obtained in another study by Gür Çetinkaya et al. (35) which analyzed 107 children with at least one SR after a Hymenoptera sting, treated with a conventional protocol. Overall, 52 (48.5%) children showed an allergic reaction during VIT: 40 (37.4%) local reactions, seven (6.5%) SR, and five (4.7%) LLR. Most local reactions occurred during the build-up phase (*p* < 0.001) and were more frequently observed with wasp VIT (*p* = 0.047). Regarding SR, these were mostly mild-moderate (grades 1–3 according to Mueller classification) and occurred more frequently for honeybee VIT (*p* = 0.031). Of the 68 children whose parents completed a follow-up survey, a re-sting during or after VIT occurred in 33 (48.5%) subjects, and 24 (72.7%) showed reactions that were more frequently local. Pre-existing asthma was the only risk factor for LLR and SR in a multivariate analysis (*p* = 0.016).

### Clustered and semi-rush protocols

In a study by Konstantinou et al., 54 children with at least one anaphylactic reaction to Hymenoptera venom were treated with a modified clustered protocol, consisting of a build-up phase lasting roughly 5 weeks and reaching a maintenance dose of 50 mcg (28). The maintenance dose was given every 4 weeks for the first year, then every 5 weeks for the second and third years, and every 6 weeks for the last two years. One child was lost to follow-up. Almost all remaining children (52/53) tolerated the protocol without side effects except for mild injection site reactions. Two children (3.8%) showed SR during VIT, and in these cases, the maintenance dose was increased to 100 mcg. Twenty-one (41.2%) of the 51 children who completed the modified-clustered protocol have been re-stung at least once with no SR reported, thus demonstrating the safety of clustered protocol and the efficacy even with a lower maintenance dose.

In another study by Carballada González et al., 21 children who were mostly allergic to honeybees (80.9%) and treated with a semi-rush protocol with one or two weekly injections of nine increasing doses of venom, an SR rate of 9.5% was reported (2/21 children) (36). Seven of 21 patients (33%) were re-stung after VIT, and none showed an SR, confirming the efficacy of the intervention.

### Rush and ultra-rush protocols

In a small study by Glaeser et al., three of the included 19 patients (15.8%) showed an anaphylactic reaction to rush honeybee venom immunotherapy (37). Nonetheless, all children could continue VIT with a modified up-dosing protocol and did not experience further side effects, but no conclusions can be drawn due to the small sample size of the study.

Another study by Stoevesandt et al. analyzed the safety of a 3- or 5-days rush protocol in a cohort of 71 children/adolescents and 981 adult controls (38). In this work, 5-day build-up protocols (*p* = 0.011; OR 2.64; CI 1.25–5.57) and honeybee VIT (*p* = 0.039; OR 2.25; CI 1.04–4.87) were associated with an increased risk of SR during VIT. While children usually may show a lower rate of SR than adults, in this study, SR were reported in 6.9% of children compared to 2.5% of adults (*p* = 0.046). However, all pediatric SR cases were mild/moderate (grades 1–2), and this discrepancy was attributed by the authors to the higher frequency of honeybee allergy in children (32.4%) than in adults (14.7%) (*p* < 0.001).

Two studies on different populations used the same ultra-rush protocol. Birnbaum et al. (43) showed a 10.8% rate of SR in 51 children (74.5% with yellow jacket allergy), while Nittner-Marszalska et al. (39) reported a 3.7% rate in 134 children (51.5% with honeybee allergy). In both studies, the risk of severe SR during VIT significantly increased with the severity of the first reaction. Besides honeybee allergy, no other risk factors were found to be statistically significant.

Kohli-Weisner et al. (41) also used an ultra-rush protocol in 94 children, mostly with an allergy to honeybees (65%). Among 102 desensitization procedures, 16 (16%) showed adverse effects. Most of these reactions were, however, grades 1–2 (50%), and none required adrenaline administration. Interestingly, younger children (4–8 years) had no SR, compared to older children and adolescents, who reported 18% and 19% SR, respectively.

In another modified ultra-rush protocol study in a cohort of 38 children by Steiss et al. (42) no SR was reported, and most children (58.1%) showed no reactions at all. In a more recent retrospective study by the same group, performed on a larger cohort, the same modified ultra-rush protocol was demonstrated to be safe as well, despite a 2.2% rate of mild SR (40).

Analyzing the various published studies, it can be seen that some defined ultra-rush protocols are really similar compared to others labeled as rush.

### Comparison between different protocols

In a study by Johnston et al., ultra-rush and conventional protocols were compared in a small cohort of 14 pediatric honeybee-allergic children. Six received ultra-rush honeybee VIT and showed a lower rate of SR (16%) compared with the conventional group (25%) while requiring fewer injections, hospital visits, and saving travel distance (44).

Similarly, in a study by Confino-Cohen et al., rush and conventional protocols were compared in children (45). Eighty-four out of 127 children were treated with rush VIT and compared to those treated with conventional VIT. Slightly more SR (23.2% vs. 19.0%) were reported during the build-up phase in the conventional group, but this difference was not statistically significant. No severe SR was reported, and the need for adrenaline was more frequent in the conventional group (9.3% vs. 4.7%, not statistically significant). Nonetheless, a significantly higher proportion of children in the rush group reached the maintenance dose compared to the conventional protocol (98.8% vs. 90.7%). Authors conclude that rush protocols are safe in childhood and more efficient than conventional protocols in terms of compliance, despite being less frequently prescribed in the United States.

### Omalizumab pretreatment

Droitcourt et al. (46) have pretreated three male teenagers undergoing VIT with omalizumab. All had severe anaphylaxis with the Hymenoptera sting (two by honeybee and one by wasp) and reported SR during the build-up phase of a rush VIT protocol. Pretreatment with omalizumab 2 and 4 weeks before VIT onset allowed to complete build-up and reach the maintenance dose without SR.

## Conclusion

In children, the occurrence of SR with conventional VIT may be similar to other protocols, although conventional VIT appears to be more frequently chosen in clinical practice than other protocols. However, greater severity of reported allergic reactions may not be excluded with faster protocols. It is also important to underline the difficulty in comparing published studies on different regimens, where there seems to be high variability among different experiences.

To date, it is still difficult to recommend one VIT protocol over another in children. The choice of protocol must always be discussed with caregivers, even though sometimes, they may be dependent on local expertise. Future extensive data coming from high-quality studies, including, e.g., the potential role of component-resolved diagnostics, of the molecular identification of allergens in the extracts used for VIT or of specific biomarkers may help tailor the choice of protocol on patients in a precision medicine perspective.

## References

[1] Wood RA Camargo CAJ Lieberman P Sampson HA Schwartz LB Zitt M Anaphylaxis in America: the prevalence and characteristics of anaphylaxis in the United States. J Allergy Clin Immunol. (2014) 133(2):461–7. 10.1016/j.jaci.2013.08.016 24144575

[2] Alvarez-Perea A Ameiro B Morales C Zambrano G Rodríguez A Guzmán M Anaphylaxis in the pediatric emergency department: analysis of 133 cases after an allergy workup. J Allergy Clin Immunol Pract. (2017) 5(5):1256–63. 10.1016/j.jaip.2017.02.011 28389303

[3] Sturm GJ Varga E-M Roberts G Mosbech H Bilò MB Akdis CA EAACI Guidelines on allergen immunotherapy: hymenoptera venom allergy. Allergy. (2018) 73(4):744–64. 10.1111/all.13262 28748641

[4] Bilò MB Pravettoni V Bignardi D Bonadonna P Mauro M Novembre E Hymenoptera venom allergy: management of children and adults in clinical practice. J Investig Allergol Clin Immunol. (2019) 29(3):180–205. 10.18176/jiaci.0310 30183660

[5] Golden DBK Kagey-Sobotka A Norman PS Hamilton RG Lichtenstein LM. Outcomes of allergy to insect stings in children, with and without venom immunotherapy. N Engl J Med. (2004) 351(7):668–74. 10.1056/NEJMoa022952 15306668

[6] Lange J Cichocka-Jarosz E Marczak H Lis G Brzyski P Nowak-Węgrzyn A. Natural history of hymenoptera venom allergy in children not treated with immunotherapy. Ann Allergy Asthma Immunol. (2016) 116(3):225–9. 10.1016/j.anai.2015.12.032 26945496

[7] Braun. Notes on desensitisation of a patient hypersensitive to bee stings. Bee World. (1926) 8(10):159–159. 10.1080/0005772x.1926.11096103

[8] Busse W Reed C Lichtenstein LM. Protection following honey-bee venom immunotherapy in a case of bee sting anaphylaxis. J Allergy Clin Immunol. (1974) 53:104.

[9] Hunt KJ Valentine MD Sobotka AK Benton AW Amodio FJ Lichtenstein LM. A controlled trial of immunotherapy in insect hypersensitivity. N Engl J Med. (1978) 299(4):157–61. 10.1056/NEJM197807272990401 78446

[10] Lichtenstein LM Valentine MD Sobotka AK. A case for venom treatment in anaphylactic sensitivity to hymenoptera sting. N Engl J Med. (1974) 290(22):1223–7. 10.1056/NEJM197405302902204 4133096

[11] Chipps BE Valentine MD Kagey-Sobotka A Schuberth KC Lichtenstein LM. Diagnosis and treatment of anaphylactic reactions to hymenoptera stings in children. J Pediatr. (1980) 97(2):177–84. 10.1016/s0022-3476(80)80470-7 7400882

[12] Dhami S Zaman H Varga E-M Sturm GJ Muraro A Akdis CA Allergen immunotherapy for insect venom allergy: a systematic review and meta-analysis. Allergy. (2017) 72(3):342–65. 10.1111/all.13077 28120424

[13] Schuberth KC Lichtenstein LM Kagey-Sobatka A Szklo M Kwiterovich KA Valentine MD. Epidemiologic study of insect allergy in children. II. Effect of accidental stings in allergic children. J Pediatr. (1983) 102(3):361–5. 10.1016/S0022-3476(83)80649-0 6827407

[14] Valentine MD Schuberth KC Kagey-Sobotka A Graft DF Kwiterovich KA Szklo M The value of immunotherapy with venom in children with allergy to insect stings. N Engl J Med. (1990) 323(23):1601–3. 10.1056/NEJM199012063232305 2098016

[15] Boyle RJ Oude Elberink J. Venom immunotherapy for preventing allergic reactions to insect stings. Cochrane Database Syst Rev. (2012) 10:10. 10.1002/14651858.CD008838.pub2 PMC8734599 23076950

[16] Müller UR Ring J. When can immunotherapy for insect sting allergy be stopped? J Allergy Clin Immunol Pract. (2015) 3(3):324–8. 10.1016/j.jaip.2014.11.018 25956311

[17] Fiedler C Miehe U Treudler R Kiess W Prenzel F. Long-Term follow-up of children after venom immunotherapy: low adherence to anaphylaxis guidelines. Int Arch Allergy Immunol. (2017) 172(3):167–72. 10.1159/000458707 28380475

[18] Hoffman DR Jacobson RS. Allergens in hymenoptera venom XII: how much protein is in a sting? Ann Allergy. (1984) 52(4):276–8. PMID: 6711914

[19] Schumacher M Tveten M Egen N. Rate and quantity of delivery of venom from honeybee stings. J Allergy Clin Immunol. (1994) 93(5):831–5. 10.1016/0091-6749(94)90373-5 8182223

[20] Roumana A Pitsios C Vartholomaios S Kompoti E Kontou-Fili K. The safety of initiating hymenoptera immunotherapy at 1 μg of venom extract. J Allergy Clin Immunol. (2009) 124(2):379–81. 10.1016/j.jaci.2009.05.026 19560804

[21] Golden DBK. Venom immunotherapy. Immunol Allergy Clin North Am. (2020) 40(1):59–68. 10.1016/j.iac.2019.09.002 31761121

[22] Goldberg A Confino-Cohen R. Maintenance venom immunotherapy administered at 3-month intervals is both safe and efficacious. J Allergy Clin Immunol. (2001) 107(5):902–6. 10.1067/mai.2001.114986 11344360

[23] Goldberg A Confino-Cohen R. Effectiveness of maintenance bee venom immunotherapy administered at 6-month intervals. Ann Allergy Asthma Immunol. (2007) 99(4):352–7. 10.1016/S1081-1206(10)60552-2 17941283

[24] Simioni L Vianello A Bonadonna P Marcer G Severino M Pagani M Efficacy of venom immunotherapy given every 3 or 4 months: a prospective comparison with the conventional regimen. Ann Allergy Asthma Immunol. (2013) 110(1):51–4. 10.1016/j.anai.2012.09.014 23244659

[25] Ruëff F Wenderoth A Przybilla B. Patients still reacting to a sting challenge while receiving conventional hymenoptera venom immunotherapy are protected by increased venom doses. J Allergy Clin Immunol. (2001) 108(6):1027–32. 10.1067/mai.2001.119154 11742283

[26] Reisman R Livingston A. Venom immunotherapy: 10 years of experience with administration of single venoms and 50 μg maintenance doses. J Allergy Clin Immunol. (1992) 89(6):1189–95. 10.1016/0091-6749(92)90304-K 1607552

[27] Houliston L Nolan R Noble V Pascoe E Hobday J Loh R Honeybee venom immunotherapy in children using a 50-μg maintenance dose. J Allergy Clin Immunol. (2011) 127(1):98–9. 10.1016/j.jaci.2010.07.031 20864150

[28] Konstantinou GN Manoussakis E Douladiris N A 5-year venom immunotherapy protocol with 50 μg maintenance dose: safety and efficacy in school children. Pediatr Allergy Immunol. (2011) 22(4):393–7. 10.1111/j.1399-3038.2010.01137.x 21235631

[29] Golden DBK. Rush venom immunotherapy: ready for prime time? J Allergy Clin Immunol Pract. (2017) 5(3):804–5. 10.1016/j.jaip.2016.12.031 28483322

[30] van der Zwan JC Flinterman J Jankowski IG Kerckhaert JA. Hyposensitisation to wasp venom in six hours. Br Med J. (1983) 287(6402):1329–31. 10.1136/bmj.287.6402.1329 6416398 PMC1549515

[31] Golden DBK Demain J Freeman T Graft D Tankersley M Tracy J Stinging insect hypersensitivity: a practice parameter update 2016. Ann Allergy Asthma Immunol. (2017) 118(1):28–54. 10.1016/j.anai.2016.10.031 28007086

[32] Diwakar L Noorani S Huissoon AP Frew AJ Krishna MT. Practice of venom immunotherapy in the United Kingdom: a national audit and review of literature. Clin Exp Allergy. (2008) 38(10):1651–8. 10.1111/j.1365-2222.2008.03044.x 18727621

[33] Martínez-Cañavate A Tabar AI Eseverri JL Martín F Pedemonte-Marco C. An epidemiological survey of hymenoptera venom allergy in the spanish paediatric population. Allergol Immunopathol. (2010) 38(5):259–62. 10.1016/j.aller.2010.02.004 20580150

[34] Albuhairi S El Khoury K Yee C Schneider L Rachid R. A twenty-two-year experience with hymenoptera venom immunotherapy in a US pediatric tertiary care center 1996–2018. Ann Allergy Asthma Immunol. (2018) 121(6):722–728.e1. 10.1016/j.anai.2018.08.002 30102964

[35] Gür Çetinkaya P Esenboğa S Uysal Soyer Ö Tuncer A Şekerel BE Şahiner ÜM. Subcutaneous venom immunotherapy in children. Ann Allergy Asthma Immunol. (2018) 120(4):424–8. 10.1016/j.anai.2018.01.015 29625665

[36] Carballada González FJ Crehuet Almirall M Manjón Herrero A De la Torre F Boquete París M. Hymenoptera venom allergy: characteristics, tolerance and efficacy of immunotherapy in the paediatric population. Allergol Immunopathol. (2009) 37(3):111–5. 10.1016/S0301-0546(09)71721-5 19769842

[37] Glaeser A Müller C Bode S. Anaphylactic reactions in the build-up phase of rush immunotherapy for bee venom allergy in pediatric patients: a single-center experience. Clin Mol Allergy. (2022) 20(1):4. 10.1186/s12948-022-00170-3 35488298 PMC9052590

[38] Stoevesandt J Hosp C Kerstan A Trautmann A. Safety of 100 µg venom immunotherapy rush protocols in children compared to adults. Allergy Asthma Clin Immunol. (2017) 13(1):32. 10.1186/s13223-017-0204-y 28706538 PMC5506672

[39] Nittner-Marszalska M Cichocka-Jarosz E Małaczyńska T Kraluk B Rosiek-Biegus M Kosińska M Safety of ultrarush venom immunotherapy: comparison between children and adults. J Investig Allergol Clin Immunol. (2016) 26(1):20–47. 10.18176/jiaci.0006 27012015

[40] Steiss JO Lindemann H. Safety of modified ultra-rush venom immunotherapy in children. J Allergy Ther. (2013) 04(02):134. 10.4172/2155-6121.1000134

[41] Köhli-Wiesner A Stahlberger L Bieli C Stricker T Lauener R. Induction of specific immunotherapy with hymenoptera venoms using ultrarush regimen in children: safety and tolerance. J Allergy. (2012) 2012:1–5. 10.1155/2012/790910 PMC3140184 21804830

[42] Steiss JO Jödicke B Lindemann H. A modified ultrarush insect venom immunotherapy protocol for children. Allergy Asthma Proc. (2006) 27(2):148–50. PMID: 16724635

[43] Birnbaum J Ramadour M Magnan A Vervloet D. Hymenoptera ultra-rush venom immunotherapy (210 min): a safety study and risk factors. Clin Exp Allergy. (2003) 33(1):58–64. 10.1046/j.1365-2222.2003.01564.x 12534550

[44] Johnston N Belcher J Preece K Bhatia R. Review of venom immunotherapy at a regional tertiary paediatric centre. J Paediatr Child Health. (2022) 58(7):1228–32. 10.1111/jpc.15964 35416342

[45] Confino-Cohen R Rosman Y Goldberg A. Rush venom immunotherapy in children. J Allergy Clin Immunol Pract. (2017) 5(3):799–803. 10.1016/j.jaip.2016.10.011 27914814

[46] Droitcourt C Ponvert C Dupuy A Scheinmann P Abou-Taam R de Blic J Efficacy of a short pretreatment with omalizumab in children with anaphylaxis to hymenoptera venom immunotherapy: a report of three cases. Allergol Int. (2019) 68(2):268–9. 10.1016/j.alit.2018.09.003 30342888

